# Anesthesia Management of a 20-Month-Old Patient with Giant Unilateral Wilms Tumor

**DOI:** 10.1155/2015/487219

**Published:** 2015-02-26

**Authors:** Nune Matinyan, Alexander Saltanov, Leonid Martynov, Anatolij Kazantsev

**Affiliations:** N. N. Blokhin Cancer Research Center, Pediatric Oncology and Hematology Research Institute, Moscow 115478, Russia

## Abstract

Wilms tumour (WT) (or nephroblastoma) is one of the most common malignant kidney tumors in children. On subsequent stages clinically it is often characterized by abdominal hypertension syndrome, which, in turn, leads to development of respiratory insufficiency. Other symptoms comprise renal deficiency, hypertension, and abnormalities of hemostasis and hemogram. Treatment includes rounds of preoperative chemotherapy and subsequent surgery. We report a case of perioperative management for nephrectomy in 20-month-old patient with a giant unilateral WT. The complexity of anesthesia was determined by the size of tumor, increased intra-abdominal pressure, respiratory deficiency, and hypercoagulation.

## 1. Introduction

WT incidence accounts for 1/10,000 children. On presentation typical symptoms may include abnormally large abdomen, fever, nausea and vomiting, hypertension, tachycardia, and tachypnea. Diagnostic evaluation includes ultrasound and computed tomography (CT) scans, complete blood count (CBC), biochemistry, urinalysis, and coagulation tests. Treatment consists of rounds of polychemotherapy with subsequent surgery. Anesthesia management in these patients is complicated by respiratory and renal insufficiency, coagulation disorders, and anemia [1]. We report a successful case of anesthesia management for nephrectomy in 20-month-old patient with a giant unilateral nephroblastoma. The complexity of the perioperative period was determined by the size of tumor, increased intra-abdominal pressure, respiratory deficiency, and hypercoagulation. Lung-protective ventilation and administration of minimal doses of opioids due to the epidural anesthesia implementation provided surgical team with optimal conditions for secure tumor extraction, followed by extubation and awakening on the operating table. Postoperative analgesia was performed using an epidural catheter. After 3 days in intensive care unit patient was transferred to Oncology Department for adjuvant postsurgery chemotherapy.

## 2. Case Report

**Figure 1:**
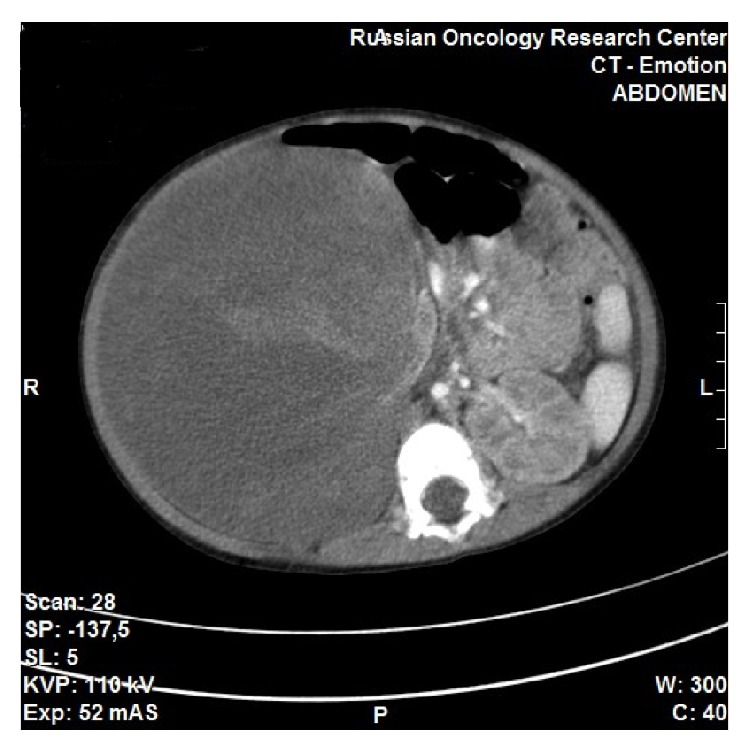
Preoperative abdominal computed tomography: right kidney subtotally replaced by tumor.

A 20-month-old female was admitted to our hospital with complaints of palpable unilateral abdominal mass, abdominal pain, fever, tachycardia, and tachypnea. Abdominal CT scans revealed right kidney subtotally replaced by tumor with dimensions 100 × 120 × 140 mm. Lungs CT revealed bilateral multilobar pneumonia, multiple atelectasis, and hypoventilation. Diagnostic evaluation was confirmed by punction biopsy: nephroblastoma cells. After antibacterial therapy and 3 rounds of chemotherapy without convincing tumor reduction, which took 3 weeks, decision to perform the surgery for tumor resection was made. Day prior to surgery physical exam revealed tachycardia 164 beats per minute, tachypnea 60 breaths per minute, and blood pressure 135/65 mmHg. Abdominal CT scans revealed right kidney subtotally replaced by tumor with dimensions 150 × 130 × 220 mm (Figure 1). Lab results showed hemoglobin: 85 g/L, platelet count: 864.000/mcL, total leucocyte count: 15,9 × 10^9^/L, hypoproteinemia: 48 g/L, and hypoalbuminemia: 21.0 g/L. Patient was staged ASA V due to progressing respiratory deficiency and lack of pathomorphosis after polychemotherapy; she was not expected to survive without the operation.

Patient presented to the operating theater in sitting position with tachycardia (160 beats per minute), tachypnea (63 breaths per minute). and SpO_2_ 89%. ECG, capnometry and capnography, BIS monitoring, and noninvasive blood pressure monitoring were started. Peripheral intravenous catheter was installed; two unsuccessful attempts to place a radial artery catheter for invasive arterial pressure monitoring were made.

Forced air warming and fluid warmer were used in order to maintain intraoperative normothermia.

Induction of anesthesia was started in Fowler (reverse Trendelenburg) position with sevoflurane 8%, nasogastric tube was installed, fentanyl 50 *μ*g (4.5 *μ*g/kg) and rocuronium 7 mg (0.63 mg/kg) were administered. After 150 s direct laryngoscopy and endotracheal intubation with 4.0 cuffed endotracheal tube were performed, and transesophageal Doppler monitoring was established. Ventilation was straitened after intubation, SatO_2_ 89–92% with FiO_2_ 100%.

Considering the large size of the tumor and the possibility of blood loss due to involvement of major blood vessels, vena subclavia sinistra and vena femoralis dextra were catheterized under ultrasound guidance for intraoperative fluid administration. Continuous infusion with rate of 1.6 mg/hour of rocuronium was established for intraoperative neuromuscular blockade.

Epidural space on T_9_-T_10_ level was identified using loss-of-resistance technique and epidural catheter 19 G was installed. A test dose of 0.5 mL lidocaine 2% was injected into the catheter to exclude subarachnoidal placement. To maintain intraoperative analgesia continuous infusion 1,6 mL per hour (0.15 mL/kg/hr) of triple component mixture of ropivacaine (2 mg/mL), fentanyl (2 *μ*g/mL), and epinephrine (2 *μ*g/mL) for intraoperative analgesia was started before the incision [2]. Fentanyl 50 *μ*g (4.5 *μ*g/kg) was administered just before the incision; additional opioids administration was not required during subsequent surgery.

Anesthesia was maintained with sevoflurane 1.8–2.3%. Patient was ventilated with pressure control mode.

Complete blood count and blood gases analyses were performed before the incision: hemoglobin: 76 g/L, platelet count: 607.000/mcL, total leucocyte count: 13,3 × 10^9^/L, pH: 7.474, PCO_2_: 30.2 mmHg, and PO_2_: 76 mmHg. Blood pressure was 75/30 mmHg and heart rate was 110 beats per minute.

**Figure 2:**
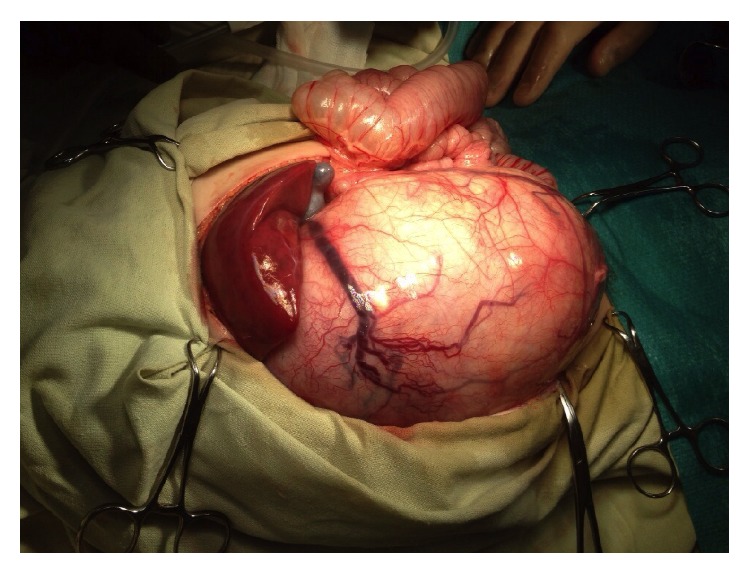
Partial tumor mobilization.

The abdomen was explored through a midline incision. As expected, solid mass occupying the whole right half of the abdomen was visualized. The medial margin of the tumor was soldered with greater curvature of the stomach; the lower margin of the tumor was soldered with right adnexa. Stomach, right salpinx, and ovary were separated from the tumor. After incision and partial mobilization of the tumor (Figure 2), ventilation, saturation, and cardiac output were gradually improving. Cardiac output increased from 1.0 to 1.4 L/min according to transesophageal Doppler. Patient's mechanical ventilation was greatly facilitated and pressure control mode with Pinsp 14 and PEEP 5 was selected. At this point we could adjust FiO_2_ from 100% to 50% and patient was positioned supine (from Fowler). Inferior vena cava was separated from the tumor; right renal vein and right renal artery were mobilized and crossed. The upper margin of the tumor was soldered with the right dome of the diaphragm, grown into the S6 segment of the liver and into the right adrenal gland. Mobilization of the tumor was accompanied with resection of the right dome of the diaphragm, S6 segment of the liver, and right adrenal gland. Nephrectomy on the right side was performed. Biopsy of two para-aortic lymph nodes was taken. After installation of the drains the surgical wound was closed. Patient was awakened and extubated on the operating table. Surgery time was 340 min.

**Figure 3:**
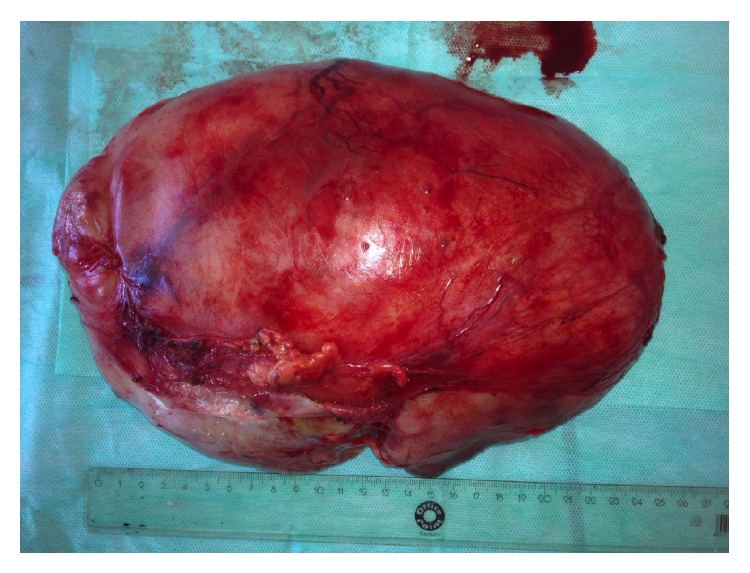
Resected specimen.

Resected specimen weighted 2200 gr (Figure 3); subsequent histological analysis matched the diagnosis of WT (nephroblastoma).

Total intraoperative IV fentanyl consumption was 1.5 *μ*g/kg/hour.

Intraoperative blood loss was 150 mL, diuresis: 100 mL. Intraoperative infusion comprised 200 mL of red blood cells, 300 mL of balanced isotonic sodium solution, and 80 mL of gelatin 4% solution.

Blood gases analysis was performed at the end of the surgery, pH: 7.3, PCO_2_: 42.2 mmHg, and PO_2_: 214 mmHg.

Postoperative course was favorable and uncomplicated, after 3 days in intensive care unit patient was transferred to Oncology Department for postsurgery chemotherapy. The patient remained fit at a follow-up examination, 3 months after surgery.

## 3. Discussion

Anesthesia management of pediatric patients with large abdominal mass is always complex and challenging. Due to asymptomatic course of early stages of diseases and the need for lengthy preoperative chemotherapy surgery is often performed in later stages of the disease. Due to rapid mass growth there is no time for development of coping mechanisms and infants with WT may present with tachypnea or dyspnea, hypertension, tachycardia, hypoproteinemia, and hypercoagulation. In our case respiratory insufficiency developed due to bilateral multilobar pneumonia and hypoventilation associated with high intra-abdominal pressure.

In our case plan of anesthesia was induction with sevoflurane, endotracheal intubation, epidural catheter placement for perioperative and prolonged postoperative analgesia, and awakening and extubation on operating table [3]. Lowest possible doses of opioids were used in order not to compromise respiratory function in the early postoperative period [4].

Epidural analgesia with three-component mixture in a patient with the V ASA grade provided smooth intraoperative period without the expressed hypotension. Lipophilic adjuvant opioid fentanyl provided a quick and adequate analgesic effect at the segmental level. Low concentrations of ropivacaine provided adequate analgesia without severe neuromuscular block and hypotension. And adding epinephrine intensified analgesia due to the action on the alpha-2 adrenergic receptors and also inhibited absorption of the mixture. Resorting to epidural analgesia of this kind during emergency surgery in a patient with V ASA grade allowed not only the adequate analgesia, but also rapid recovery of vital functions in the immediate postoperative period (extubation on the operating table) [2, 3, 5].

Transesophageal dopplerography provided intraoperative preload and cardiac output monitoring. Sufficient intraoperative infusion allowed avoiding a severe drop in blood pressure at the moment of mobilization of the giant tumor.

BIS monitoring allowed administering the optimal dose of inhalational anesthetic for maintenance of anesthesia. Prompt recovery was achieved due to intraoperative normoventilation, stable hemodynamic, maintenance of normothermia, and the use of low-dose IV opioid (fentanyl 1.5 mkg/kg/hour).

In conclusion, in this successful case anesthesiologist's goal was to secure adequate perioperative anesthesia management, providing optimal conditions for the surgical team, to conduct adequate postoperative analgesia, provision of early activation, and rehabilitation.
